# Activated Stellate Cell Paracrine HGF Exacerbated Pancreatic Cancer Cell Ferroptosis Resistance

**DOI:** 10.1155/2022/2985249

**Published:** 2022-06-01

**Authors:** Qiwei Wu, Lian Song, Yaxin Guo, Sai Liu, Wenyao Wang, Huli Liu, Aihua Gong, Xiang Liao, Haitao Zhu, Dongqing Wang

**Affiliations:** ^1^Department of Medical Imaging, The Affiliated Hospital of Jiangsu University, Zhenjiang, China 212001; ^2^School of Medicine, Jiangsu University, Zhenjiang, China 212013; ^3^Department of Medical Laboratory, The Affiliated Hospital of Jiangsu University, Zhenjiang, China 212001; ^4^Central Laboratory of Radiology, The Affiliated Hospital of Jiangsu University, Zhenjiang 212001, China

## Abstract

As a refractory tumor, pancreatic carcinoma is more vulnerable to ferroptosis, a novel regulated cell death mode. However, the exact role of pancreatic stellate cells (PSCs) in pancreatic cancer ferroptosis is still unclear. Using the coculture system, we revealed that activated PSCs promote pancreatic cancer cell ferroptosis resistance. Mechanistically, activated PSCs secreted HGF, which further activated the HGF receptor, c-MET, in pancreatic cancer cells, prevented lipid peroxidation, and ultimately triggered pancreatic cancer cell ferroptosis resistance *in vitro* and *in vivo*. TCGA and GEPIA databases also revealed a strong correlation between c-MET and antiferroptosis indicators. Our study supplied the evidence for the cross-talk between activated PSCs and pancreatic cancer cells in ferroptosis, which suggested a strategy to inhibit PSC paracrine signaling for preventing pancreatic carcinoma ferroptosis resistance.

## 1. Introduction

Pancreatic ductal adenocarcinoma (PDAC) is known for its resistance to traditional therapy, with a high percentage of recurrence and a poor five-year survival rate [1]. Recently, several reports revealed that PDAC are more vulnerable to ferroptosis inducers [2]. Ferroptosis is a new type of iron-dependent programmed cell death. However, the inherent mechanisms of ferroptosis resistance in pancreatic cancer, especially the role played by stromal cells in the tumor microenvironment, deserve further investigation.

Ferroptosis is driven by iron accumulation, lipid peroxidation, and subsequent plasma membrane rupture which is distinct from other types of regulated cell death such as apoptosis and necrosis [3]. Although ferroptosis is a promising efficacy in various refractory tumors, cancer cells developed multiple resistance mechanisms to cope with ferroptosis inducers. Cancer cells have a high expression of FSP1 [4], SLC7A11 [5], and GPX4 [6] to resist ferroptosis. FSP1 functioned as an oxidoreductase that reduced coenzyme Q (CoQ) to halt lipid peroxide propagation, and GPX4 inhibited lipid peroxidation directly [7]. Most of the current researches on cancer cell ferroptosis resistance are focused on the intrinsic mechanisms. However, the influence of the extrinsic factors on cancer cell ferroptosis resistance is still unclear. Recently, studies have reported that hypoxia [8] and egregious lactate [9] help cancer cells resist ferroptosis inducers, indicating that extrinsic factors may also be involved in cancer cell ferroptosis resistance. As the most abundant stromal cells in tumor tissues, the role of cancer-associated fibroblasts (CAFs) in the process of ferroptosis is still uncovered.

Pancreatic stellate cells (PSCs) are the main source of pancreatic cancer CAFs [10], and they are closely related to the occurrence and development of PDAC. Coimplanted cancer cells and CAFs resulted in increased treatment resistance and redox status in an orthotopic xenograft model of PDAC in mice [11]. Additionally, the PSCs could interact with the cancer cells via a direct and indirect manner to support tumor nonessential amino acids and lipid biosynthesis metabolism, which further promote cancer cell survival and cell death resistance [12]. Due to the critical role of redox status and lipid biosynthesis in cancer cell ferroptosis, we speculate PSCs may function in a paracrine avenue to promote pancreatic cancer cell ferroptosis resistance.

To test this hypothesis, we used the coculture model and revealed that activated PSCs promote pancreatic cancer cell ferroptosis resistance. Activated PSCs secreted HGF, which resulted in increased c-mesenchymal-epithelial transition (c-MET) expression and enhanced antioxidant capacity in pancreatic cancer cells. Accordingly, it may be possible to combine antifibrotic drugs with ferroptosis inducers, as a new potential clinical treatment strategy, to promote refractory pancreatic carcinoma ferroptosis.

## 2. Materials and Methods

### 2.1. Cell Culture and Isolation

Mouse pancreatic cancer cell line (Panc02) was obtained from the Cell Bank of the Chinese Academy of Sciences (Shanghai, China) and cultured in Roswell Park Memorial Institute (RMPI) supplemented with 10% fetal bovine serum (FBS), penicillin (100 U/ml), and streptomycin (0.1 mg/ml).

Primary mouse pancreatic stellate cells were isolated from 6-week-old mouse pancreatic tissues according to the previous publication [13]. Briefly, pancreatic tissues were cut into 1 mm^3^ tissue blocks with sterile surgical scissors and transferred to 6-well culture plates with 5-8 fragments per well. DMEM/F-12 (1 : 1) medium containing 10% FBS, penicillin (100 U/ml), and streptomycin (0.1 mg/ml) was added, and the culture medium was changed every 2 days. All cell lines were cultured in a constant humidity incubator containing 5% CO_2_ at 37°C.

### 2.2. Cell Coculture

Millicell Hanging Cell Culture Insert system (MCHT06H48) was used as the coculture system. To verify the activation of pancreatic stellate cells by Panc02 cells, pancreatic stellate cells were inoculated in lower chambers, while Panc02 cells were inoculated in the upper chambers for 72 h. Moreover, to confirm the effect of activated PSCs on Panc02 cell ferroptosis resistance, Panc02 cells were inoculated in the lower chambers, while activated PSCs were inoculated in the upper chambers for 72 h, and then, ferroptosis inducers Erastin/RSL3 were used to treat the lower chamber cancer cells for the indicated time.

### 2.3. Cell Viability Assay

The logarithmic growth phase Panc02 cells were exposed to different conditions for 72 h, and then, the cell viability was detected with the Cell Counting Kit-8 (CCK-8, Dojindo, #CK04) reagent. CCK-8 was used according to the instructions of the reagent manufacturer. CCK-8 working solution is prepared at a ratio of CCK − 8 reagent : serum − free medium = 1 : 9. The plate was incubated for about 1-2 h at 37°C, 5% CO_2_. Absorbance (OD value) was measured at 450 nm wavelengths with a microplate reader (Biotek, #Epoch2). The percentage difference between treated and control cells was calculated to determine the relative viability of the cells.

### 2.4. Western Blot Assay

Cells were lysed on ice, and the total protein was collected. The bicinchoninic acid (BCA) method was used to determine the protein concentrations. Western blot assay was performed as previously described [14, 15]. Proteins were transferred to polyvinylidene fluoride membranes (Merck Millipore, MA, USA) via sodium dodecyl sulfate-polyacrylamide gel electrophoresis. The membranes were closed with 5% bovine serum albumin (BSA) in TBST for 1 h at room temperature and then incubated with primary antibody overnight at 4°C. Subsequently, the membranes were incubated with the second antibodies for 1 h at room temperature. The immunoreactive bands were detected via chemiluminescence (ECL Plus, Merck Millipore). Antibodies were as follows: c-MET Rabbit mAb (ABclonal, #A17366, 1 : 1000), SLC7A11/xCT Rabbit mAb (ABclonal, #A2413, 1 : 1000), GPX4 Monoclonal antibody (Proteintech, #14432-1-AP, 1 : 1000), and Anti-beta Tubulin antibody (Abcam, #ab6046, 1 : 1000). The secondary antibodies (either antirabbit or antimouse) were obtained from Thermo Fisher Scientific (Invitrogen).

### 2.5. Quantitative Real-Time Polymerase Chain Reaction (qRT-PCR) Assay

The TRIzol (Takara) reagent was used to extract total RNA from cells in different treatment groups, and the RevertAid First-Strand cDNA Synthesis Kit (Thermo, #K1622) was used to synthesize cDNA from 1 *μ*g total RNA. Then, SYBR Green master mix (Vazyme), DEPC water (Beyotime), primers, and cDNA were added into the CFX-96 system (Bio-Rad) in an appropriate proportion, and real-time PCR was performed on a QuantStudio 3 real-time PCR machine (Invitrogen). For analysis, the threshold cycle (Ct) values for each gene were normalized to those of ACTB. The primers were synthesized and desalted from GenScript, and the sequences are shown in Table 1.

### 2.6. Iron (Fe^2+^) Detection

Cellular Fe^2+^ levels were determined with the Bio Tracker™ 575 Red Fe^2+^ Dye (Merck, #SCT030). Cells were seeded in 6-well plates, and the culture supernatants were replaced by 5 *μ*mol/L Bio Tracker™ 575 Red Fe^2+^ Dye, incubated in an incubator protected from light for 60 min. Cells were collected by centrifuging at 1000 rpm for 5 min, washed 1-2 times in PBS, and resuspended in PBS. The fluorescence intensity of cells was detected by flow cytometry with the PE channel. The average fluorescence intensity of each group indicated the intracellular Fe^2+^ level.

### 2.7. Glutathione (GSH) and Malondialdehyde (MDA) Assay

The GSH and MDA contents of cells or tumor tissues after different treatments were measured using the GSH and MDA kit (Nanjing Jiancheng, #A006-2-1&A003-1-2). The cells and tumor tissues were collected, added with PBS, and ultrasonically disintegrated or homogenized. Follow the instructions of the kit, and finally, quantify the GSH and MDA content in the sample colorimetrically (OD = 405, 532 nm).

### 2.8. Lipid ROS Assay

Cellular lipid ROS levels were determined using C11-BODIPY (Invitrogen, #D3861). Cells were seeded in 6-well plates, and the culture supernatants were replaced by 2 *μ*mol/L C11-BODIPY, incubated in an incubator protected from light for 30 min. Cells were collected by centrifuging at 1000 rpm for 5 min, washed 1-2 times in PBS, and resuspended in PBS. The fluorescence intensity of cells was detected by flow cytometry with the FITC channel. The average fluorescence intensity of each group indicated the intracellular lipid ROS level.

### 2.9. RNAi and Gene Transfection

To establish stable sh-c-MET pancreatic cancer cell lines, we packaged sh-c-MET plasmids in 293T cells and concentrated them from the cell supernatant according to the instructions and then collected the supernatant lentiviral fluid. The pancreatic cancer cells were infected with the supernatant lentiviral solution, and the stable sh-MET cell lines were successfully infected cell lines screened out by puromycin. The stable cell lines were stored in RMPI medium containing 10% FBS and 0.75 *μ*g/ml puromycin for subsequent experiments. The specific plasmid sequences can be seen in Table 2.

### 2.10. ELISA Assay

To measure the content of HGF secreted by PSCs under different treatment conditions, pancreatic stellate cells were seeded in 6-well plates and exposed to different conditions when they had reached approximately 60% fusion. The cells were collected and sonicated, the HGF content was measured with the Mouse HGF ELISA Kit (Elabscience, #E-EL-M3033, Wuhan, China) according to the instructions.

### 2.11. Xenograft Tumor Models

Animal studies were approved by the Committee on the Use of Live Animals for Teaching and Research of Jiangsu University. 5-6-week-old female C57BL/6 mice were purchased from Cavens Laboratory Animals Co., Ltd. (Changzhou, China). The mice were kept under standard conditions.

Thirty-six C57BL/6 mice were randomly divided into six groups. Panc02 cells (2 × 10^5^) were injected into the right dorsal subcutis of the mice with or without pancreatic stellate cells (1 × 10^5^) in 60 *μ*l PBS. When the tumor volume reaches 50-100 mm^3^, the mice were treated as the following groups: DMSO (control), HGF (50 ng/kg), Piperazine Erastin (MCE, #HY-100887, USA) (30 mg/kg), and HGF (50 ng/kg)+Piperazine Erastin (30 mg/kg). Treatments were administered every two days for 14 days, and tumor size was measured and recorded along with mouse body weight.

### 2.12. Immunofluorescence (IF) Analyses

The activation of pancreatic stellate cells was detected by immunofluorescence. Briefly, isolated pancreatic stellate cells were treated with different conditions and seeded on 24-well plates. After fixation, permeabilization, and blocking, they were incubated overnight at 4°C with antimouse *α*-SMA (CST, #19245, 1 : 500) antibody. After binding the secondary antibody, it was observed and analyzed under a fluorescent microscope.

### 2.13. Immunohistochemistry (IHC) Analysis

The mouse tumor tissues were removed, fixed with 4% paraformaldehyde, and embedded in paraffin wax, and 4 *μ*m thick tissue sections were cut off for IHC analysis. Afterwards, the sections were dewaxed and then incubated with mouse primary antibody c-MET (ABclonal, #A17366, 1 : 200), at 4°C overnight. After being stained with hematoxylin, the sections were sealed with neutral resin, observed, and analyzed under an Olympus microscope.

### 2.14. Patient Selection

The Gene Expression Profiling Interactive Analysis (GEPIA) database which contains 350 samples was used to analyze the differential expression levels of c-MET genes in pancreatic cancer tissues and normal tissues, as well as the relationship between the expression levels of c-MET and the overall survival rate of pancreatic cancer patients. The Cancer Genome Atlas (TCGA) database (http://tcga.xenahubs.net/download/TCGA.PAAD.sampleMap/HiSeqV2.gz) which includes 183 pancreatic carcinoma patient specimens was also utilized to analyze the relationship between c-MET and several antiferroptosis indicators. High and low groups were defined as above and below the mean, respectively.

### 2.15. Statistical Analysis

All data were presented as the mean ± standard error of the mean (SEM). Statistical analysis was performed using Prism 7.0 software. The differences between groups were analyzed using Student's *t* test, one-way analysis of variance (ANOVA), or two-way ANOVA. *P* < 0.05 was considered to have statistically significant difference. All biology experiments were repeated at least three times, and the remaining experiments were repeated at least two times.

## 3. Results

### 3.1. Activated PSCs Promoted Pancreatic Cancer Cell Ferroptosis Resistance

To access the role of PSCs in pancreatic cancer cell ferroptosis sensitivity, the isolated primary PSCs were cocultured with Panc02 cells (Figure S1A). Compared to the control PSCs, cocultured PSCs (Co-PSCs) displayed polygonal and flat morphology and a reduced number of red clusters representing lipid droplets (Figures S1B and S1C), which is according to the previous publication [16]. Moreover, western blot, qRT-PCR, and immunofluorescence revealed that the expression level of *α*-SMA was significantly upregulated in Co-PSCs (Figures S1D, S1E, and S1F). These results suggested that isolated primary PSCs were activated in the presence of pancreatic cancer cells.

**Figure 1:**
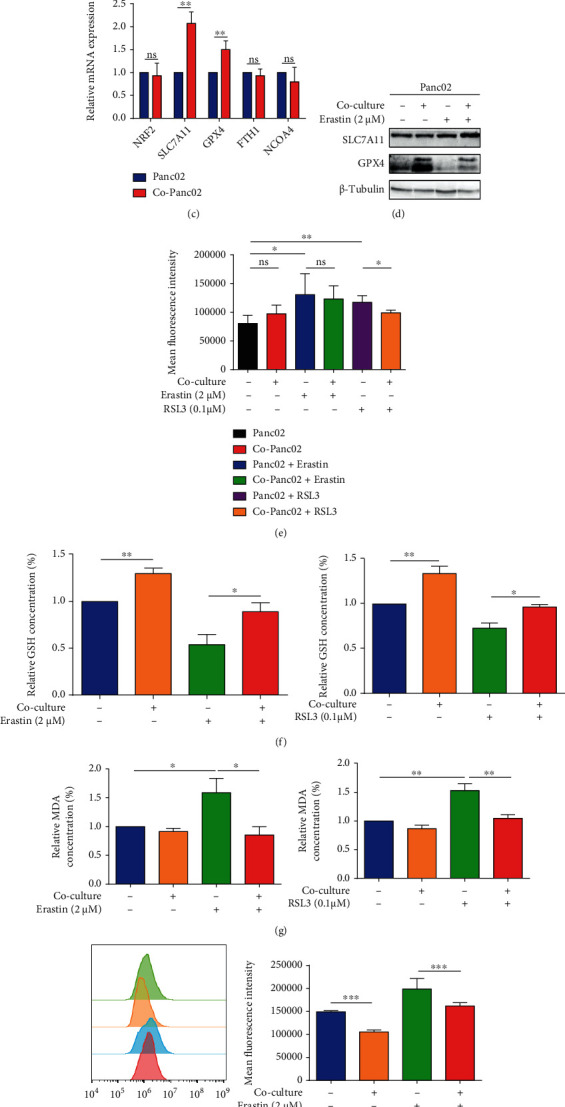
The activated PSCs promote pancreatic cancer cell ferroptosis resistance. (a) A proposed model illustrating the coculture system of Panc02 cells. (b) Panc02 cells were treated with different concentrations of Erastin (0, 0.5, and 1 *μ*M) or RSL3 (0, 0.05, and 0.1 *μ*M) for 72 hours under normal or coculture and Fer-1 conditions. Cell viability was measured by CCK-8 kits. (c) qRT-PCR analysis of the mRNA expression levels of ferroptosis indicators (NRF2, SLC7A11, GPX4, FTH1, and NCOA4) in Panc02 cells under normal and coculture conditions. ACTB mRNA expression was detected as a loading control. (d) Western blot analysis of the protein expression levels of ferroptosis indicators (SLC7A11 and GPX4) in Panc02 treated with DMSO, coculture, Erastin (2 *μ*M), and coculture+Erastin (2 *μ*M). *β*-tubulin expression was detected as a loading control. (e–h) Panc02 cells were treated with DMSO, coculture, Erastin (2 *μ*M)/RSL3 (0.1 *μ*M), and coculture+Erastin (2 *μ*M)/RSL3 (0.1 *μ*M). Iron (Fe^2+^) level of Panc02 was evaluated by flow cytometry (e). The relative GSH (f) and MDA (g) concentrations of Panc02 were analyzed. Lipid ROS level of Panc02 was evaluated by flow cytometry (h). Fer-1 represents ferrostatin-1. Co-Panc02 represents Panc02 cells which were cocultured with activated PSCs. Experiments were repeated three times, and data were expressed as the mean ± SEM. ^∗^*P* < 0.05, ^∗∗^*P* < 0.01, and ^∗∗∗^*P* < 0.001.

We next assessed the ferroptosis sensitivity of pancreatic cancer cells with or without activated PSCs. Panc02 were cocultured with or without activated PSCs, followed by treatment with Erastin (an inhibitor of system Xc^−^) or RSL3 (an inactivated agent of GPX4) (Figure 1(a)). Compared with the control group, Erastin and RSL3 could significantly induce cell death, which could be rescued by ferrostatin-1 (Fer-1), indicating ferroptosis. Additionally, Erastin- or RSL3-induced cell death was reversed in the presence of activated PSCs (Figure 1(b)).

In the presence of activated PSCs, GPX4 expression was significantly increased at mRNA and protein levels in Panc02, as well as SLC7A11 at the mRNA level, whereas the mRNA levels of FTH1 and NCOA4 (iron metabolism indicators) were not significantly altered (Figures 1(c) and 1(d)). Moreover, Fe^2+^ levels were also assessed, and the results showed that Erastin and RSL3 elevated Panc02 cellular Fe^2+^ levels. In contrast, activated PSCs had no significant effect on the Fe^2+^ content of Panc02 cells (Figure 1(e)). Since system Xc^−^ synthesizes GSH through importing cysteine and is responsible for maintaining redox homeostasis, we asked whether activated PSCs affected the GSH concentration in Panc02 cells. The GSH level was inhibited and the MDA level (an end product of lipid peroxidation) was increased in Panc02 cells following Erastin and RSL3 treatment, which were counteracted by coculturing with activated PSCs (Figures 1(f) and 1(g)). Meanwhile, coculturing with activated PSCs effectively inhibited the accumulation of lipid ROS induced by ferroptosis inducers (Figure 1(h)). To conclude, activated PSCs enhanced the resistance to ferroptosis of pancreatic cancer.

### 3.2. HGF Secreted by Activated PSCs Mediated Ferroptosis Resistance in Pancreatic Cancer Cells

**Figure 2:**
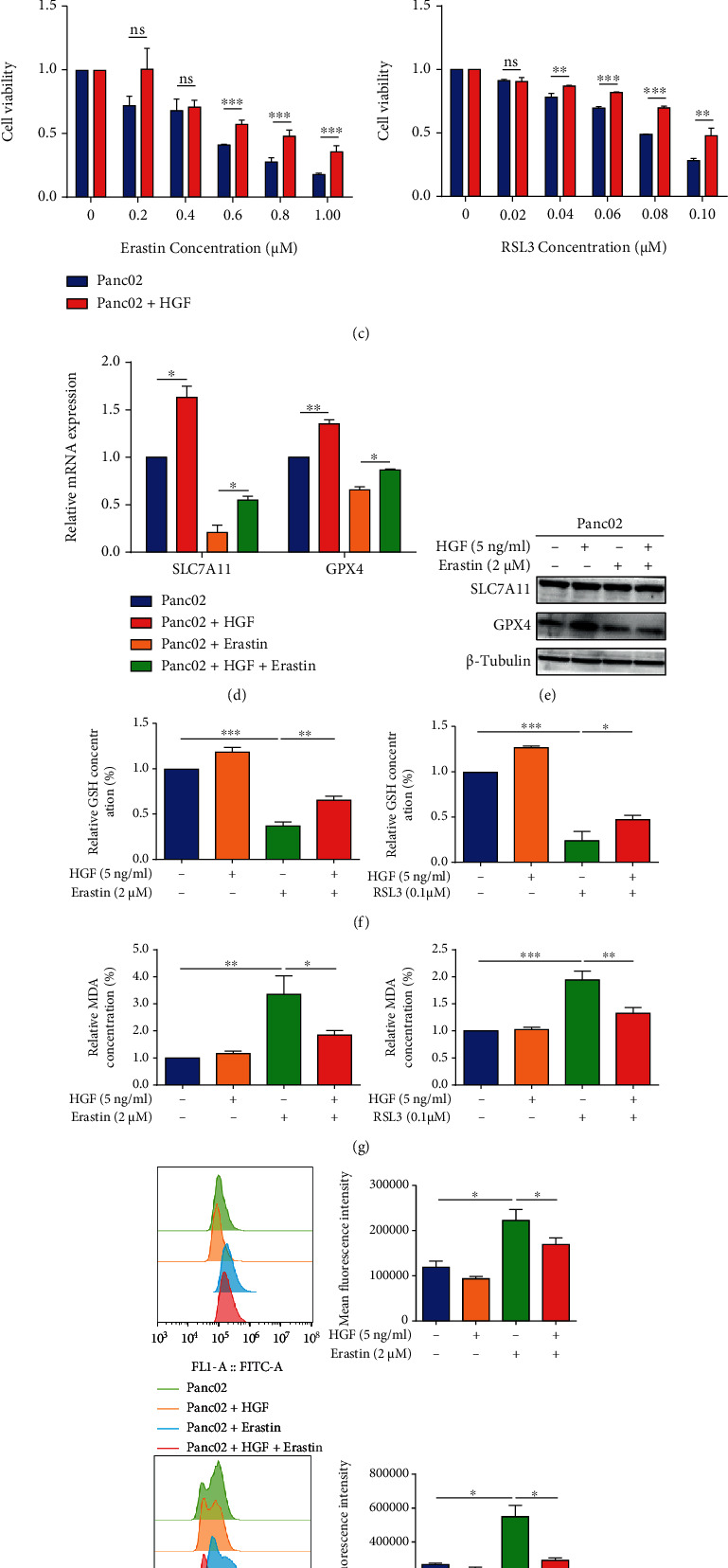
HGF secreted by activated PSCs mediated ferroptosis resistance in pancreatic cancer cells. (a) qRT-PCR analysis of mRNA expression levels of multiple secreted factors (CXCL-16, TNF-*α*, CCL7, HGF, TGF-*β*, PDGF, CCL2, VEGF, SDF1-A, IGF-1, IL-6, b-FGF, and CCL5) in PSCs under normal or coculture conditions. (b) ELISA-based analysis of the HGF concentration in PSCs under normal and coculture conditions. (c) Panc02 cells were treated with HGF (5 ng/ml) and different concentrations of Erastin (0, 0.2, 0.4, 0.6, 0.8, and 1 *μ*M) or RSL3 (0, 0.02, 0.04, 0.06, 0.08, and 0.1 *μ*M) for 72 hours. Cell viability was measured by CCK-8 kits. (d, e) qRT-PCR and western blot analysis of the mRNA and protein expression levels of ferroptosis indicators (SLC7A11 and GPX4) in Panc02 cells treated with DMSO, HGF (5 ng/ml), Erastin (2 *μ*M), or HGF (5 ng/ml)+Erastin (2 *μ*M). ACTB mRNA expression was detected as a loading control for qRT-PCR. *β*-tubulin expression was detected as a loading control for western blot. (f–h) Panc02 cells were treated with DMSO, HGF (5 ng/ml), Erastin (2 *μ*M)/RSL3 (0.1 *μ*M), or HGF (5 ng/ml)+Erastin (2 *μ*M)/RSL3 (0.1 *μ*M). The relative levels of GSH (f) and MDA (g) of Panc02 were assayed by test kits, and the relative levels of Panc02 lipid ROS were detected by flow cytometry (h). Experiments were repeated three times, and data were expressed as the mean ± SEM. ^∗^*P* < 0.05, ^∗∗^*P* < 0.01, and ^∗∗∗^*P* < 0.001.

Activated PSCs regulate pancreatic cancer cell biology depending on various cytokines secreted. We next evaluated the expression of these cytokines at the mRNA level in Co-PSCs. CCL7, HGF, CCL2, SDF-1*α*, IGF-1, and CCL5 were upregulated in Co-PSCs with HGF as the most significantly upregulated (Figure 2(a)). The ELISA assay further verified that HGF secreted by cocultured PSCs was significantly increased (Figure 2(b)).

To further validate the role of activated PSC-derived HGF in Panc02 ferroptosis, Panc02 cells were cultured with HGF (5 ng/ml) and then treated with different concentrations of ferroptosis inducers. Compared to the normal control group, Panc02 cancer cells treated with HGF enhanced cell viability in the presence of ferroptosis inducers (Figure 2(c)). Then, we treated Panc02 with the following condition: (1) DMSO, (2) HGF (5 ng/ml), (3) Erastin (2 *μ*M) or RSL3 (0.1 *μ*M), and (4) HGF (5 ng/ml)+Erastin (2 *μ*M)/RSL3 (0.1 *μ*M). Compared to the DMSO group, HGF treatment increased SLC7A11 and GPX4 expression at the mRNA level as well as GPX4 at the protein level (Figures 2(d) and 2(e)). HGF reversed the reduction of GSH and accumulation of MDA induced by Erastin or RSL3 (Figures 2(f) and 2(g)). Furthermore, HGF+Erastin/RSL3 groups showed a suppressed lipid ROS level in Panc02 cells compared to Erastin/RSL groups (Figure 2(h)). These data implied that HGF secreted by activated PSCs promoted ferroptosis resistance in pancreatic cancer cells.

### 3.3. Inhibition of c-MET Sensitized Pancreatic Cancer Cells to Ferroptosis with the Presence of Activated PSCs

**Figure 3:**
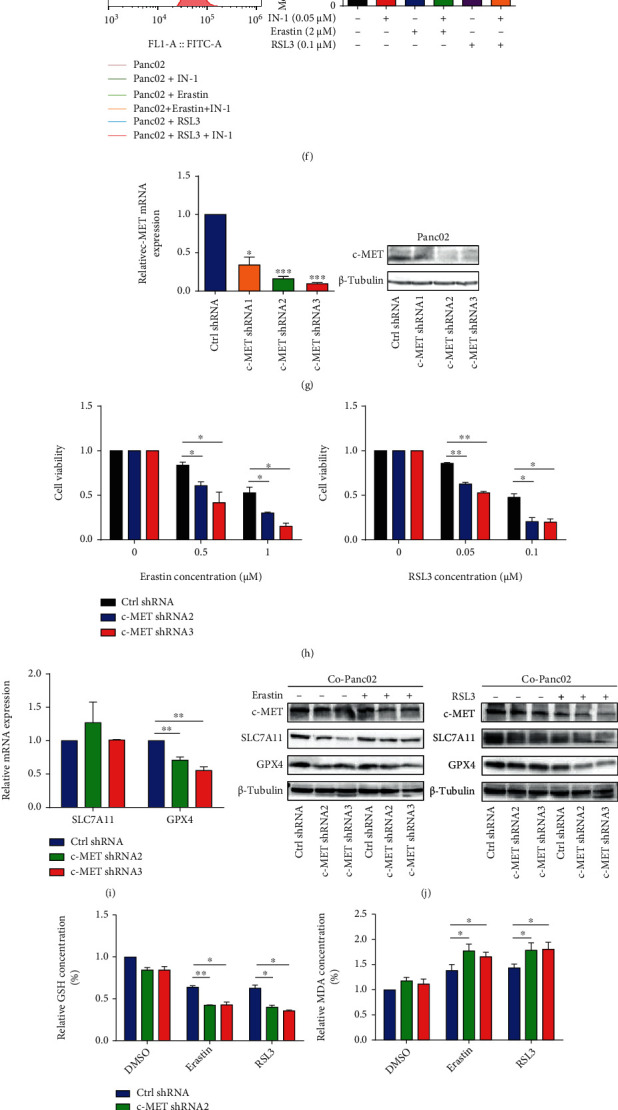
Inhibition of c-MET sensitizes pancreatic cancer cells to ferroptosis with the presence of activated PSCs. (a, b) Panc02 cells treated with DMSO, c-MET-IN-1, Erastin, Erastin+IN-1, RSL3, and RSL3+IN-1 cultured under normal or coculture conditions. Representative images showing the induction of cell death in Panc02 cells (a) and cell viability was measured by CCK-8 kits (b). (c–f) Panc02 cells were cocultured with activated PSCs and then treated with DMSO, IN-1 (0.05 *μ*M), Erastin (2 *μ*M), Erastin (2 *μ*M)+c-MET-IN-1 (0.05 *μ*M), RSL3 (0.1 *μ*M), and RSL3 (0.1 *μ*M)+c-MET-IN-1 (0.05 *μ*M). Western blot analysis of the protein expression levels of c-MET, SLC7A11, and GPX4 in Co-Panc02 (c). *β*-tubulin expression was detected as a loading control. The relative GSH (d) and MDA (e) concentrations of Co-Panc02 were analyzed; lipid ROS level of Co-Panc02 was evaluated by flow cytometry (f). (g) qRT-PCR and western blot analysis of shRNA-mediated knockdown of c-MET mRNA and protein levels in Panc02 cells. ACTB mRNA expression was detected as a loading control for qRT-PCR. *β*-tubulin expression was detected as a loading control for western blot. (h) Co-Panc02 cells (Ctrl shRNA, c-MET shRNA1, and c-MET shRNA2) were treated with different concentrations of Erastin (0, 0.5, and 1 *μ*M) or RSL3 (0, 0.05, and 0.1 *μ*M) for 72 hours. Cell viability was measured by CCK-8 kits. (i) qRT-PCR analysis of SLC7A11 and GPX4 mRNA expression in Panc02 (Ctrl shRNA) and c-MET knockdown Panc02 (c-MET shRNA1, c-MET shRNA2, and c-MET shRNA3). (j–l) Co-Panc02 cells (Ctrl shRNA, c-MET shRNA1, and c-MET shRNA2) were treated with Erastin (2 *μ*M) or RSL3 (0.1 *μ*M). Western blot analysis of the protein expression levels of c-MET, SLC7A11, and GPX4 in Co-Panc02. *β*-tubulin expression was detected as a loading control (j). The relative GSH and MDA concentrations of Co-Panc02 were analyzed (k); lipid ROS level of Co-Panc02 was evaluated by flow cytometry (l). IN-1 represents c-MET-IN-1. Experiments were repeated three times, and data were expressed as the mean ± SEM. ^∗^*P* < 0.05, ^∗∗^*P* < 0.01, and ^∗∗∗^*P* < 0.001.

c-Mesenchymal-epithelial transition (c-MET), an HGF receptor, has been shown to play a decisive role in therapy resistance in various cancers. To further confirm the role of the HGF/c-MET axis in Panc02 ferroptosis resistance, we added c-MET inhibitor, c-MET-IN-1, to the coculture system. c-MET-IN-1 significantly enhanced the sensitivity of pancreatic cancer cells to ferroptosis and reduced cell survival (Figures 3(a) and 3(b)). c-MET-IN-1 combined with ferroptosis inducers (Erastin and RSL3) reduced the protein expression levels of GPX4 (Figure 3(c)). c-MET-IN-1 also significantly promoted the reduction of GSH and the accumulation of MDA and lipid ROS induced by ferroptosis inducers (Figures 3(d)–3(f)).

In addition to inhibited c-MET at the pharmacological level, two stable c-MET knockdown pancreatic cancer cell clones (c-MET shRNA2 and shRNA3) with higher silencing efficiency (Figure 3(g)) were selected for the subsequent experiments. Erastin and RSL3 induced more c-MET-knockdown pancreatic cancer cell death under coculture conditions or in the presence of HGF (Figure 3(h) and Figure S2A). Knockdown c-MET downregulated the GPX4, rather than SLC7A11 at the mRNA and protein level in Panc02 cells (Figures 3(i) and 3(j) and Figure S2B). Additionally, knockdown of c-MET reduced GSH content and increased MDA and lipid ROS production in activated PSC coculture and HGF-treated pancreatic cancer cells in the presence of ferroptosis inducers (Figures 3(k) and 3(l) and Figures S2C and S2D).

### 3.4. Activated PSCs Promoted Ferroptosis Resistance in Pancreatic Cancer *In Vivo*

**Figure 4:**
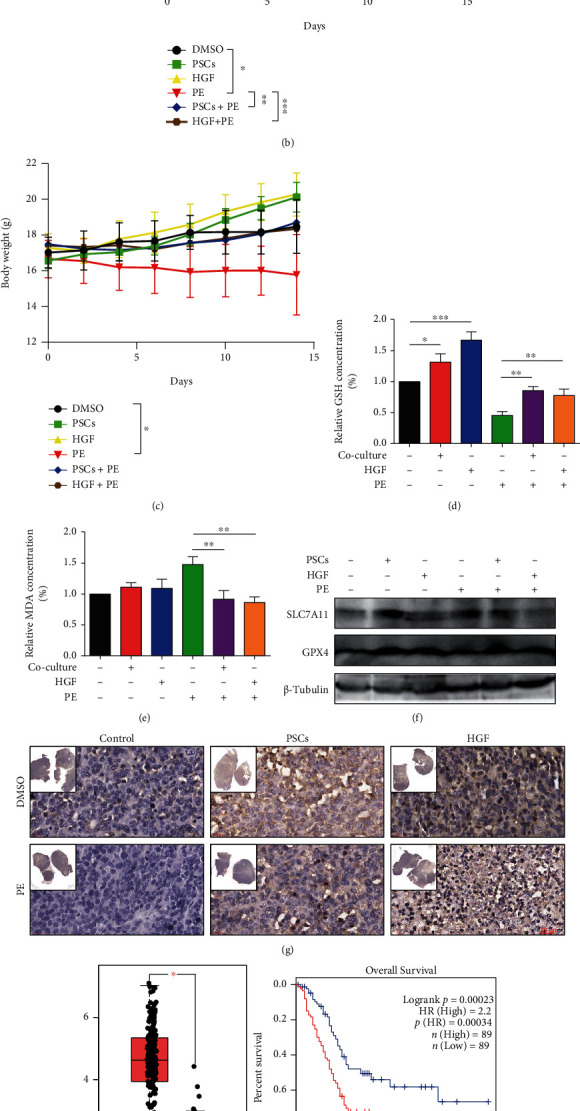
Activated PSCs promoted ferroptosis resistance in pancreatic cancer *in vivo*. (a–g) C57BL/6 mice were injected subcutaneously with Panc02 (Panc02 groups, 2 × 10^5^ cells/mouse) or Panc02+PSCs (Co-Panc02 groups, 1.5 × 10^5^ Panc02 cells + 0.5 × 10^5^ PSCs). They were divided into Panc02 groups (treated with DMSO), Co-Panc02 groups (DMSO), Panc02+H (HGF, 50 ng/i.h., every two days), Panc02+E (Piperazine Erastin, 30 mg/kg/i.h., every two days), Co-Panc02+E (Piperazine Erastin, 30 mg/kg/i.h., every two days), and Panc02+H+E (HGF, 50 ng/i.h., Piperazine Erastin, 30 mg/kg/i.h., every two days). (a) Representative photographs of isolated tumor tissues in each treatment group at day 14. (b) Tumor volume was detected every two days. (c) The body weight of mice in each treatment group was measured every two days. (d, e)D-E. The GSH (d) and MDA (e) levels in isolated tumors were assayed at day 14 after different treatments. (f) Western blot analysis of protein expression levels of ferroptosis-related indicators (SLC7A11 and GPX4) of isolated tumor tissues in each treatment group. (g) Immunohistochemistry analysis of the expression of c-MET in isolated tumor tissues. (h) The expression of c-MET in PDAC patients and normal pancreatic tissues (T: tumor; N: normal) and the correlation between the expression of c-MET and the survival of PDAC patients were analyzed with the GEPIA database. (i) Analysis of the TCGA and GEPIA databases for the correlation c-MET mRNA expression level with the ferroptosis-related indicators (NRF2, SLC7A11, and GPX4). The TCGA database result was presented by heat map: *n* = 183. PE: Piperazine Erastin. Experiments were repeated three times, and the data were expressed as the mean ± SEM. ^∗^*P* < 0.05, ^∗∗^*P* < 0.01, and ^∗∗∗^*P* < 0.001.

To further confirm our *in vitro* observations, we next investigated whether activated PSCs and HGF have a role in promoting ferroptosis resistance in pancreatic cancer *in vivo*. Panc02 with or without activated PSCs was then injected subcutaneously in C57BL/6 mice. The C57BL/6 mice were divided randomly into the following groups (*n* = 6): (1) DMSO (control), (2) PSCs, (3) HGF, (4) PE, (5) PSCs+PE, and (6) HGF+PE, and treated with the according agents every two days. Piperazine Erastin (PE) effectively inhibited the growth of subcutaneous tumors in C57BL/6 mice (Figures 4(a) and 4(b)). Compared to the control DMSO group, the PE group had a lower body weight, reduced GSH level, and increased MDA level (Figures 4(c)–4(e)). Compared to the control group, the PSC+PE and HGF+PE groups showed no significant changes in tumor volume, as well as GSH and MDA level (Figures 4(b), 4(d), and 4(e)). Compared to the PE group, PSC+PE and HGF+PE groups showed increased GSH level and decreased MDA level (Figures 4(d) and 4(e)). Simultaneously, the PSC group and HGF group also showed higher expression levels of GPX4 relative to the control group (Figure 4(f)). Consistent with *in vitro* results, PSCs and HGF were also involved in pancreatic cancer ferroptosis resistance *in vivo*.

Immunohistochemistry also further demonstrated a high expression of c-MET in tumor tissues of the PSC and HGF groups (Figure 4(g)). The GEPIA database showed that c-MET expressed a higher level in human pancreatic cancer than the normal pancreatic tissues. Moreover, the c-MET expression level was negatively correlated with the overall survival rate (Figure 4(h)). GEPIA and TCGA database analysis showed significant positive correlation of c-MET with NRF2 and SLC7A11, which are ferroptosis resistance indicators (Figure 4(i)), indicating that c-MET was involved in ferroptosis resistance.

## 4. Discussion

**Figure 5:**
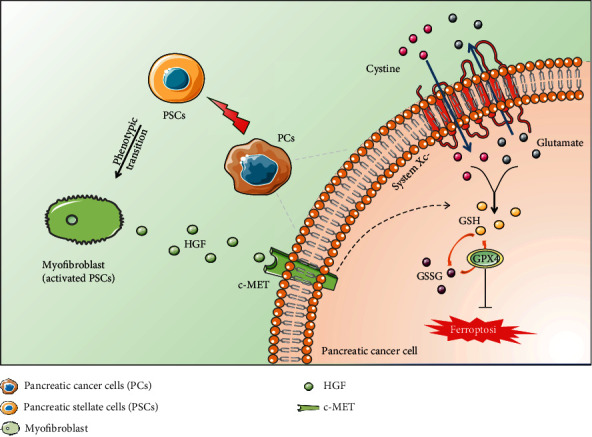
Schematic representation of the mechanisms by which the activated PSC paracrine HGF mediated pancreatic cancer cell ferroptosis resistance.

In this study, we demonstrated that pancreatic cancer cells were able to activate the quiescent PSCs, creating a tumor microenvironment that was conducive to their survival. The activated PSCs could secrete HGF, which enhanced the antioxidant capacity of pancreatic cancer cells, thereby resisting ferroptosis (Figure 5). In this regard, the HGF/c-MET axis had a vital role in the defense of pancreatic cancer cells against ferroptosis.

Pancreatic cancer was daunting due to it being extremely aggressive and difficult to treat [17]. Pancreatic cancer cells have been shown to develop resistance from their highly dynamic tumor microenvironment, defend against drug-killing effects, and promote their own progression [18]. More and more studies have focused on the pancreatic cancer microenvironment to find new therapeutic approaches. Fibrous tissue hyperplasia was the most prominent feature of the pancreatic cancer microenvironment, manifested by excessive fibrosis and extracellular matrix deposition [19]. Activated PSCs could secrete large amounts of extracellular matrix and cytokines, mediating the fibrosis of the pancreatic cancer microenvironment to regulate the progression of pancreatic cancer [20]. Our experiments demonstrated that quiescent PSCs could be activated by pancreatic cancer cells and that activated PSCs could in turn enhance the ferroptosis resistance of pancreatic cancer cells. Through a regulatory network coconstructed with pancreatic cancer cells, activated PSCs promoted pancreatic cancer development, metastasis, drug resistance, and immune escape and were important targets for pancreatic cancer microenvironment regulation [21].

As a novel mode of cell death, ferroptosis is characterized by the iron-dependent accumulation of lipid peroxidation to lethal levels, which is often accompanied by depletion of GSH [22]. In this study, we found that activated PSCs were able to secrete HGF in large quantities and increase the GSH content through the HGF/c-MET axis *in vitro* and *in vivo*. GSH is an antioxidant in the form of a tripeptide that acts as a synergistic molecule for selenium-dependent GPX4 and assists GPX4 in reducing lipid hydroperoxides, thus mediating ferroptosis resistance in pancreatic cancer cells [23]. Meanwhile, activated PSCs had less effect on the iron (Fe^2+^) content in pancreatic cancer cells. We speculated that PSCs affect the ferroptosis sensitivity of pancreatic cancer cells mainly by influencing their antioxidant capacity rather than iron metabolism, which needs to be confirmed by further researches. It was demonstrated that Licochalcone D could induce ROS-dependent apoptosis in lung cancer cells by inhibiting c-MET, suggesting that c-MET is closely related to the redox system [24]. Our primary results also implied that the HGF/c-MET axis affects the antioxidant capacity of pancreatic cancer cells and thus the ferroptosis sensitivity of the cells, which is according to a previous publication. However, the exact interconnection between deserves our deeper investigation. Moreover, inhibition of c-MET could sensitize ferroptosis, which indicated that the combination of ferroptosis inducer and c-MET inhibitors may achieve a satisfactory therapeutic effect, and it needs to be further validated in clinical trials. Additionally, reversing activated PSCs back to a quiescent state might also sensitize pancreatic cancer ferroptosis. It has been indicated that ATRA could keep PSCs in a quiescent state and reduce pancreatic fibrosis [25]. Combining ATRA with ferroptosis inducers may be able to mitigate the effects of the dense microenvironment of pancreatic cancer on treatment resistance [26] and provide a therapeutic effect. To conclude, inhibiting the activation of PSCs or the expression of c-MET in tumor cells in combination with ferroptosis inducers may reduce the dose of ferroptosis inducers and achieve better therapeutic effects.

Additionally, the development of cancers is a holistic process. In previous studies, too much emphasis has been placed on the molecular mechanisms of ferroptosis resistance in cancer cells themselves [27] while the effects of other stromal cells on cancer cells was neglected. In this study, we selected PSCs, which are most abundant in the pancreatic cancer tumor microenvironment, to observe their effects on ferroptosis susceptibility of pancreatic cancer cells. In addition, PSCs have also been found to influence immune cells and promote immune escape of tumors in a multifaceted way [28]. It should always be borne in mind that the tumor process is a holistic event, and we should treat the development of tumorigenesis from a macroperspective.

In summary, this study indicated that pancreatic cancer cells could activate pancreatic stellate cells, promote their secretion of HGF, enhance the antioxidant capacity of pancreatic cancer cells, and mediate ferroptosis resistance in pancreatic cancer. It implied a potential therapeutic option for the treatment of pancreatic cancer with a view to the microenvironment of pancreatic cancer fibrosis. However, this hypothesis needs to be confirmed by further researches for better treatment of pancreatic cancer.

## Figures and Tables

**Table 1:** Sequences of primers.

Name	Direction	Sequence (5′-3′)
NRF2-mouse	Forward	CAGCATAGAGCAGGACATGGAG
	Reverse	GAACAGCGGTAGTATCAGCCAG
SLC7A11-mouse	Forward	CTTTGTTGCCCTCTCCTGCTTC
	Reverse	CAGAGGAGTGTGCTTGTGGACA
GPX4-mouse	Forward	CCTCTGCTGCAAGAGCCTCCC
	Reverse	CTTATCCAGGCAGACCATGTGC
c-MET-mouse	Forward	TCACTATCTACCTGTTGCAAGG
	Reverse	CAGCATTTTAGCATCACTTCGT
CXCL16-mouse	Forward	CTGGAAGTTGTTCTTGTGATCG
	Reverse	CTGCAACTGGAACCTGATAAAG
CCL2-mouse	Forward	TTTTTGTCACCAAGCTCAAGAG
	Reverse	TTCTGATCTCATTTGGTTCCGA
TNF-*α*-mouse	Forward	ATGTCTCAGCCTCTTCTCATTC
	Reverse	GCTTGTCACTCGAATTTTGAGA
SDF-1*α*-mouse	Forward	TCTGAAAATCCTCAACACTCCA
	Reverse	CAGGTACTCTTGGATCCACTTT
b-FGF-mouse	Forward	AGTTGTGTCTATCAAGGGAGTG
	Reverse	CATTGGAAGAAACAGTATGGCC
IL-6-mouse	Forward	CTCCCAACAGACCTGTCTATAC
	Reverse	CCATTGCACAACTCTTTTCTCA
HGF-mouse	Forward	ACCTACAGGAAAACTACTGTCG
	Reverse	TGCATTCAACTTCTGAACACTG
IGF-1-mouse	Forward	GAGGGGCTTTTACTTCAACAAG
	Reverse	TACATCTCCAGTCTCCTCAGAT
TGF-*β*-mouse	Forward	CCAGATCCTGTCCAAACTAAGG
	Reverse	CTCTTTAGCATAGTAGTCCGCT
CCL7-mouse	Forward	ACAAAAGATCCCCAAGAGGAAT
	Reverse	TCTTGAAGATAACAGCTTCCCA
PDGF-mouse	Forward	GTCCAGGTGAGAAAGATTGAGA
	Reverse	GTCATGGGTGTGCTTAAACTTT
CCL5-mouse	Forward	GTATTTCTACACCAGCAGCAAG
	Reverse	TCTTGAACCCACTTCTTCTCTG
VEGF-mouse	Forward	TAGAGTACATCTTCAAGCCGTC
	Reverse	CTTTCTTTGGTCTGCATTCACA

**Table 2:** Sequences of plasmid.

Name	Sequences (5′-3′)
c-MET-shRNA1	GCTTGTTGACACATACTATGA
c-MET-shRNA2	GCATGTCAGCATCGCTCAAAT
c-MET-shRNA3	GCAGTGAATTAGTTCGCTATG

## Data Availability

The Gene Expression Profiling Interactive Analysis (GEPIA) database which contains 350 samples was used to analyze the differential expression levels of c-MET genes in pancreatic cancer tissues and normal tissues, as well as the relationship between the expression levels of c-MET and the overall survival rate of pancreatic cancer patients. The Cancer Genome Atlas (TCGA) database (http://tcga.xenahubs.net/download/TCGA.PAAD.sampleMap/HiSeqV2.gz) which includes 183 pancreatic carcinoma patient specimens was also utilized to analyze the relationship between c-MET and several antiferroptosis indicators. High and low groups were defined as above and below the mean, respectively.

## Supplementary Materials

Supplementary Materials Figure S1: pancreatic cancer cells induced PSC activation. Figure S2: inhibition of c-MET sensitized pancreatic cancer cells to ferroptosis with the presence of HGF.
