# Supplementary material for: Activated Stellate Cell Paracrine HGF Exacerbated Pancreatic Cancer Cell Ferroptosis Resistance
Source: Oxid Med Cell Longev. 2022 Jun 1;2022:2985249. doi: 10.1155/2022/2985249 (PMC9177329; doi:10.1155/2022/2985249)

**Activated stellate cells paracrine HGF exacerbated pancreatic cancer cell ferroptosis resistance**

Qiwei Wu^1^, Lian Song^1^, Yaxin Guo^1^, Sai Liu^1^, Wenyao Wang^1^, Huli Liu^1^, Aihua Gong^3^, Xiang Liao^4*^, Haitao Zhu^1,2*^, Dongqing Wang^1*^

^1^ Department of Medical Imaging, The Affiliated Hospital of Jiangsu University, Zhenjiang, China 212001

^2^Central laboratory of Radiology, Affiliated Hospital of Jiangsu University, Zhenjiang 212001, China

^3^ School of Medicine, Jiangsu University, Zhenjiang, China 212013

^4^ Department of Medical Laboratory, The Affiliated Hospital of Jiangsu University, Zhenjiang, China 212001

*Correspondence should be addressed to Dongqing Wang: [wangdongqing71@163.com](mailto:wangdongqing71@163.com); Haitao Zhu: [zhht25@163.com](mailto:zhht25@163.com); Xiang Liao: liaoxiang025@126.com.

**Figure S1. Pancreatic cancer cells induced PSCs activation.**

A. A proposed model illustrating the co-culture treatment of PSCs. B. Representative images showing the morphology of PSCs under normal and co-culture conditions. C. Oil red staining detected the lipid droplets of PSCs in normal or co-culture condition. D. Western blot analysis of protein expression levels of desmin and α-SMA in PSCs under normal and co-culture condition. E. qRT-PCR analysis of mRNA expression levels of α-SMA in PSCs under normal and co-culture condition. F. The expression level of α-SMA was evaluated by fluorescent microscope images in PSCs under normal and co-culture conditions (α-SMA, red). Experiments were repeated three times, and the date were expressed as the mean ±SEM. ^*^*P*<0.05. ^**^*P*<0.01. ^***^*P*<0.001.

**Figure S2. Inhibition of c-MET activity sensitizes pancreatic cancer cells to ferroptosis.**

A. Panc02 cells(Ctrl shRNA, c-MET shRNA1 and c-MET shRNA2) were cultured with HGF(5 ng/ml) and then treated with different concentrations of Erastin(0, 0.5, 1 μM) or RSL3(0, 0.05, 0.1 μM) for 72 hours. Cell viability was measured by CCK-8 kits. B-D. Panc02 cells(Ctrl shRNA, c-MET shRNA1 and c-MET shRNA2) were treated with HGF(5 ng/ml), Erastin(2 μM) or RSL3(0.1 μM). Western blot analysis of the protein expression levels of c-MET, SLC7A11 and GPX4 in Panc02 cells. β-tubulin expression was detected as a loading control(B). The relative GSH and MDA concentrations of Panc02 were analyzed(C), Lipid ROS level of Panc02 cells was evaluated by flow cytometry(D). Experiments were repeated three times, and the date were expressed as the mean ±SEM. ^*^*P*<0.05. ^**^*P*<0.01. ^***^*P*<0.001.

**Figure S1**


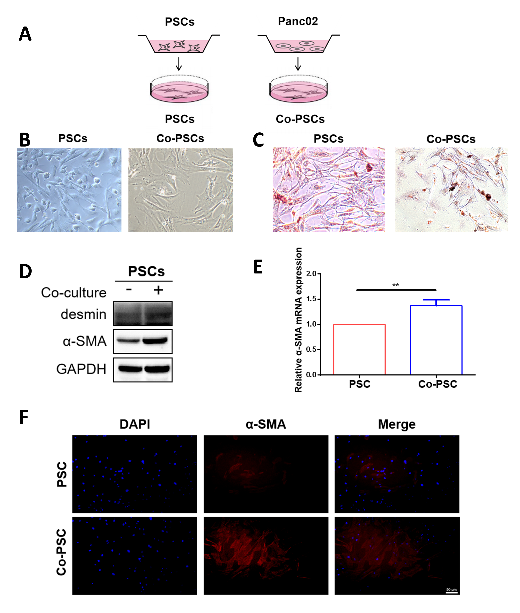


**Figure S2**


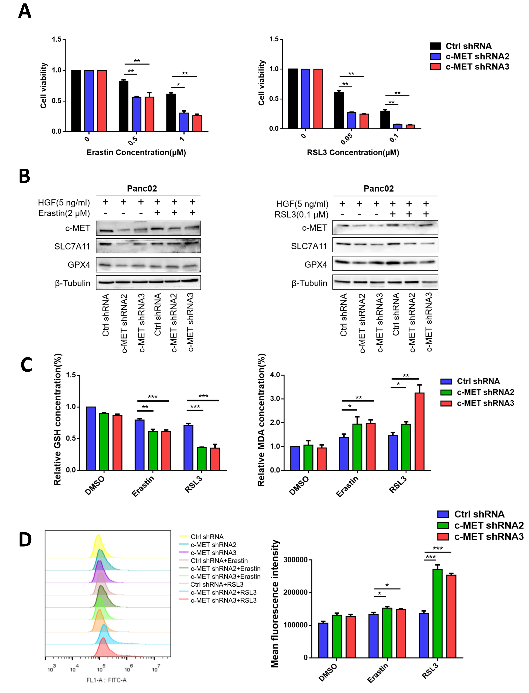

Supplement: Supplementary Materials — Figure S1: pancreatic cancer cells induced PSC activation. Figure S2: inhibition of c-MET sensitized pancreatic cancer cells to ferroptosis with the presence of HGF. [file 2985249.f1.docx]
